# The role of N-glycans and their processing in ER-to-lysosome-associated degradation of disease-causing mutant Neuroserpin

**DOI:** 10.1080/27694127.2026.2712797

**Published:** 2026-08-06

**Authors:** Carolin Hoefner, Ilaria Fregno, Maurizio Molinari

**Affiliations:** a Università della Svizzera italiana, Institute for Research in Biomedicine, Bellinzona, Switzerland; b Department of Biology, Swiss Federal Institute of Technology, Zurich, Switzerland; c School of Life Sciences, École Polytechnique Fédérale de Lausanne, Lausanne, Switzerland

**Keywords:** Conformational disease, ERLAD, ERphagy, Neuroserpin, *N*-glycosylation

## Abstract

Most proteins synthesized in the endoplasmic reticulum (ER) are covalently modified upon addition of pre-assembled oligosaccharides to side chains of asparagine (N) residues. Processing of *N*-linked oligosaccharides by ER-resident glucosidases, mannosidases and glucosyltransferases determines the fate of the associated polypeptides. Terminally glucose residues are removed from *N*-glycans to interrupt the engagement of ER-resident glucose-binding chaperones and promote secretion of native polypeptides. Mannose residues are removed to target terminally misfolded proteins for dislocation across the ER membrane and clearance by the cytoplasmic ubiquitin proteasome system (ER-associated degradation, ERAD). Recent evidence highlights the role of persistent *N*-glycan glucosylation as a signal that promotes ER lectins-driven segregation of misfolded proteins in ER subdomains that are eventually delivered to endolysosomal compartments for ER-to-Lysosome-Associated Degradation (ERLAD). Here we show that the polymerization-prone Portland variant of Neuroserpin (NS_PL) associated with familial encephalopathy with NS inclusion bodies (FENIB) is a client of the ERLAD machinery. Its lysosomal clearance relies on the LC3-dependent delivery branch of ERLAD involving the lectin chaperone Calnexin (CNX), the ERphagy receptor FAM134B and the SNARE protein Syntaxin17 (STX17), which is engaged upon persistent glucosylation of the NS_PL oligosaccharide linked at the asparagine residue at position 321.

## Introduction

The ER is the main biosynthetic organelle of nucleated cells, producing lipids, oligosaccharides, and a large fraction of the cellular proteome [1]. Most proteins synthesized in the ER are modified by *N*-linked glycosylation. *N*-glycosylation is a critical post-translational modification in Eukarya and Archaea, where a pre-assembled sugar chain (glycan) is attached to the side chain of an asparagine residue in an asparagine-any amino acid-serine/threonine (*N*-X-S/T) consensus sequence. *N*-glycosylation is operated co- or post-translationally by the oligosaccharyltransferase (OST) complex associated with the protein translocation machinery at the ER membrane [2]. *N*-glycans are composed of 3 glucose, 9 mannose, and 2 *N*-acetylglucosamine residues. Their sequential processing by ER-resident glucosidases, mannosidases, and glucosyltransferases determines the fate of the associated polypeptide, which is either retained in the ER to complete the folding program, secreted upon achievement of the native structure, or eventually degraded if folding fails [3]. Despite a folding program assisted by high concentrations of molecular chaperones and folding enzymes, up to one third of newly synthesized proteins fail to reach the native state [4]. To maintain a healthy proteome and ensure cellular homeostasis, terminally misfolded proteins are efficiently eliminated either by ER-associated degradation (ERAD) or by ER-to-Lysosome-Associated Degradation (ERLAD) [5–8]. Engagement of the ERAD machinery relies on slow removal of terminal mannose residues from *N*-linked glycans of terminally misfolded polypeptides that facilitates retro-translocation across the ER membrane, polyubiquitylation, and subsequent proteasomal degradation [9–11]. Misfolded proteins that fail to enter the ERAD pathways, enter instead ERLAD programs a collection of selective lysosomal quality control pathways that deliver ER subdomains containing misfolded proteins to acidic degradative organelles or extract misfolded proteins from the ER and transport them to lysosomal compartments. Some ERphagy-dependent ERLAD pathways rely on cycles of removal and re-addition of terminal glucose residues from misfolded proteins’ *N*-linked glycans that prolong the association with ER-resident lectin chaperones, followed by segregation into specialized ER subdomains for lysosomal removal [12,13]. The ERphagy-dependent delivery of misfolded polypeptides from the ER to the degradative compartments via ERLAD proceeds through three distinct routes [6]: (i) macro-ERphagy, in which the ER subdomains containing misfolded polypeptides are engulfed by autophagosomes that eventually fuse with endolysosomes to generate the degradative autolysosomes [14]; (ii) micro-ERphagy, involving direct capture of ER subdomains or ER exit sites by endolysosomes [15]; (iii) LC3-mediated vesicular transport, where ER-derived vesicles or ER subdomains containing the misfolded proteins to be removed from cells fuse with lysosomes [12] (thoroughly reviewed in [6]).

Neuroserpin (NS) is a neuronal serine protease inhibitor that regulates tissue plasminogen activator activity and supports synaptic function [16,17]. This soluble glycoprotein is synthesized in the ER of neurons. After proper folding, NS traffics through the Golgi apparatus and is secreted into the extracellular space [18]. Mutations in the SERPINI1 gene may introduce amino acid mutations in the NS polypeptide that destabilize the serpin fold, may promote aggregates formation and, if aggregates’ clearance is inefficient, may result in intraluminal accumulation of the mutant polypeptide in soluble or insoluble toxic form. This is linked to a hereditary neurodegenerative disease called familial encephalopathy with Neuroserpin inclusion bodies (FENIB) [18,19]. Here, we investigate the intracellular fate of the Portland mutation of NS (NS_PL). NS_PL is characterized by the replacement of the serine (S) residue at position 52 in the polypeptide sequence with an arginine (R, S52R, pink in Figure 1A). NS_PL is one of the aggregation-prone NS variants associated with FENIB [16,18–20]. Previous studies showed that clearance from the ER of this folding-defective polypeptide relies on the intervention of both the ubiquitin-proteasome system and autophagy [21]. The autophagic clearance of NS_PL was pharmacologically inhibited in cells exposed to Bafilomycin A1 (BafA1) to inactivate lysosomal hydrolases and by genetic inactivation of autophagy upon deletion of ATG5 [21]. We first confirmed these data to then establish in molecular detail the lysosomal clearance pathway that ensures the removal of NS_PL aggregates from the ER lumen. Our study shows that terminally misfolded NS_PL is an ERLAD client whose lysosomal clearance requires glucose processing of the *N*-glycan at position 321 and involves the lectin chaperone CNX, the ERphagy receptor FAM134B, the SNARE protein STX17 and is dependent on LC3 lipidation.

**Figure 1.**
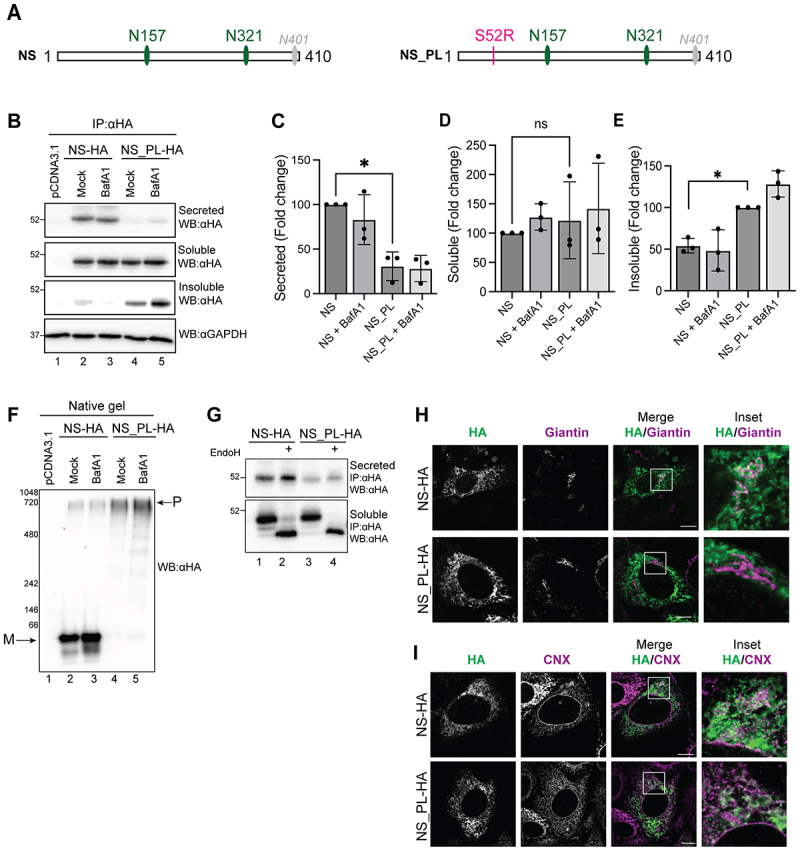
The Portland mutation hampers folding, secretion, and results in soluble and insoluble aggregates. (A) The three potential N-glycosylation sites of NS and NS_PL are shown in the schematics. Numbers refer to the position in the amino acid sequence. The N401 site is not glycosylated. The S52R mutation of NS_PL is shown. (B) HEK293 cells were transiently transfected with NS-HA or NS_PL-HA and mock-treated or exposed to 100 nM BafA1. NS-HA and NS_PL-HA were immunoisolated with anti-HA antibodies from distinct cellular fractions (secreted, detergent-soluble, and detergent-insoluble). Proteins were separated in SDS-PAGE and revealed by immunoblotting. (C) Quantification of the secreted fraction of B, *N* = 3, unpaired t-test with Welch’s correction, *p < 0.05. (D) Same as C for the detergent soluble fraction, ns *p* > 0.05. E) Same as C for the detergent insoluble fraction * *p* < 0.05. (F) Same as (B) where proteins in the detergent-soluble intracellular fraction were separated in native gels. Monomers (lanes 2, 3) and polymers (lanes 4, 5) are shown with arrows. (G) EndoH sensitivity assay of the glycoproteins immunoisolated from the cell media or the detergent soluble intracellular fraction. (H) CLSM analyses in WT MEF. NS-HA and NS_PL-HA (green) with the Golgi marker Giantin (magenta). Scale bars 10 µm. (I) Same as (H) with the ER marker CNX.

## Results

### The Portland mutation enhances NS misfolding and deposition in insoluble aggregates

Firstly, we monitored the intracellular fate of HA-tagged versions of NS or NS_PL transiently expressed in Human Embryonic Kidney (HEK)293 cells. Twelve hours after transfection, the cell culture medium was replaced with fresh medium, which was collected 6 h later to compare secretion of NS (Figure 1B, upper panel, Secreted, lanes 2, 3) with secretion of NS_PL (lanes 4, 5) as assessed by immunoblotting with anti-HA antibodies. Transfected cells were detergent-solubilized as described in the Methods section. The intracellular NS (lanes 2, 3) and NS_PL (lanes 4, 5) were monitored by immunoblotting in the detergent soluble (Figure 1B, second panel, Soluble) and in the detergent-insoluble fraction (Figure 1B, third panel, Insoluble). The mutation in NS_PL substantially inhibited the secretion of the ectopically expressed polypeptide (Figure 1B, upper panel, lanes 2 *vs*. 4, Figure 1C). In the detergent-soluble intracellular fraction, we detected comparable abundance for both variants (Figure 1B, second panel, lanes 2 *vs*. 4, Figure 1D). However, analysis of these fractions in native gels revealed that the detergent-soluble NS is monomeric (M, Figure 1F, lane 2), whereas the detergent-soluble NS_PL is in high molecular weight complexes (P, Figure 1F, lane 4), as expected from its propensity to polymerize [22]. Moreover, in contrast to NS (Figure 1B, third panel, lane 2, Figure 1E), NS_PL shows a marked accumulation in the detergent-insoluble fraction (Figure 1B, third panel, lane 4, Figure 1E).

To confirm that the NS and NS_PL harvested from the cell culture media is actively secreted from cells, we performed an endoglycanase H (EndoH) assay. EndoH cleaves *N*-glycans of ER-localized glycoproteins but fails to cleave the glycans of polypeptides actively secreted from cells via the Golgi complex. These protein-bound oligosaccharides are in fact processed by Golgi enzymes to become EndoH-resistant [23]. When subjected to the EndoH assay, both the NS and NS_PL harvested from the cell culture media do not change their electrophoretic mobility. Thus, their *N*-glycans are EndoH-resistant testifying the secretion of the glycoproteins via the Golgi complex (Figure 1G, upper panel, lanes 1 *vs*. 2 for NS and lanes 3 *vs*. 4 for NS_PL). In contrast, the polypeptides immuno-isolated from the cells’ lysate are EndoH-sensitive and showed enhanced electrophoretic mobility upon EndoH treatment (Figure 1G, second panel, lanes 1 *vs*. 2 and 3 *vs*. 4). Notably, a small fraction of intracellular NS was EndoH-resistant (Figure 1G, second panel, lanes 1 *vs*. 2), consistent with the rapid transit of this polypeptide in the Golgi Complex during secretion as testified by its co-localization with the Golgi marker Giantin in confocal laser scanning microscopy (CLSM, Figure 1H, upper panel). Consistent with previous data showing that autophagic pathways contribute to the clearance of NS_PL aggregates [21], inhibition of lysosomal activity with BafA1 led to the accumulation of both soluble and insoluble NS_PL aggregates (Figure 1B,F).

### NS_PL is delivered to LAMP1-positive endolysosomes for clearance

The propensity of misfolded NS_PL to form aggregates (Figure 1F, lanes 4–5) and the accumulation of the protein in the insoluble fraction upon inactivation of the lysosomal activity in cells exposed to BafA1 (Figure 1B, third panel, lanes 4–5) led us to check whether NS_PL is delivered within LAMP1-positive degradative endolysosomes for clearance. To this end, the subcellular distribution of NS_PL was examined in wild‑type mouse embryonic fibroblasts (MEF) in the absence (Figure 2A) or in the presence (Figure 2B) of BafA1, which inhibits lysosomal hydrolases thus resulting in accumulation of lysosomal clients in the lumen of LAMP1-positive degradative compartments [25,26]. In cells with functional endolysosomes, NS_PL co-localized with the ER marker protein calnexin (CNX) (Figure 1I) and it was not found within LAMP1-positive compartments (Figure 2A). The substantial accumulation of NS_PL within LAMP1-positive endolysosomes upon inactivation of their degradative activity in cells exposed to BafA1 (Figure 2B, quantification in Figure 2I) confirms that NS_PL is cleared by endolysosomal degradation and thereby identifies NS_PL as a client of the ERLAD machinery.

**Figure 2.**
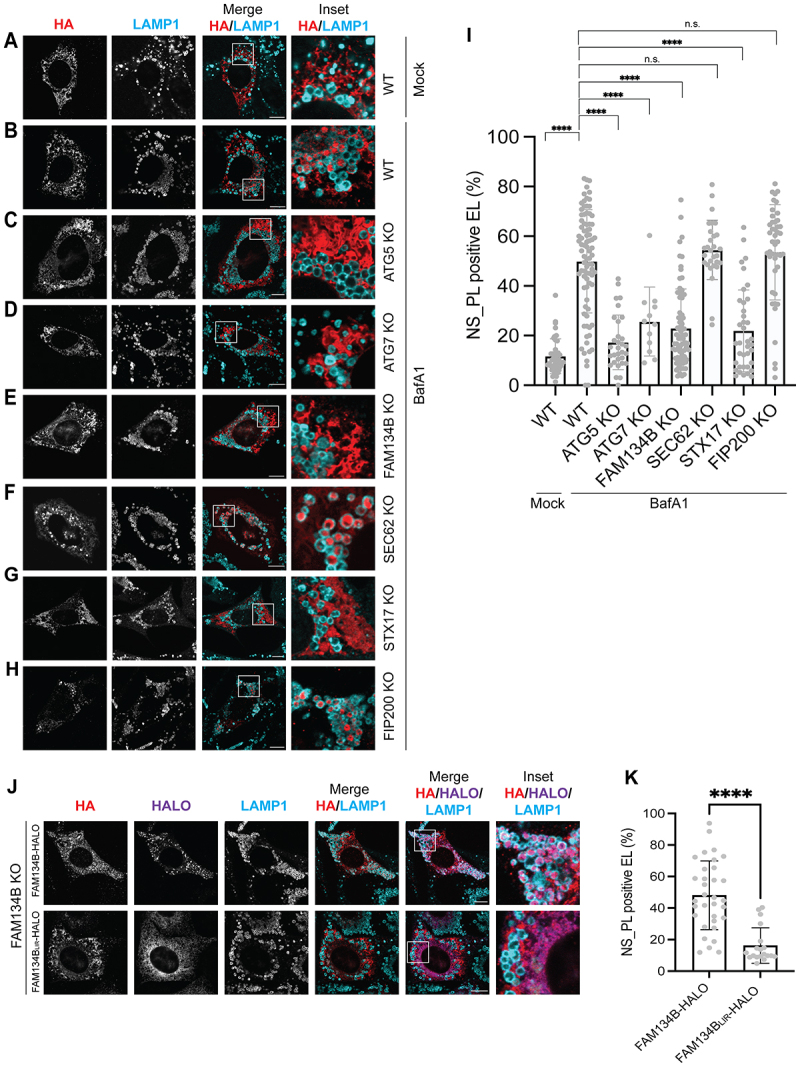
Monitoring the delivery of NS_PL to LAMP1-positive endolysosomes for degradation. (A) CLSM analyses in WT MEF. NS_PL-HA (red) in LAMP1-positive endolysosomes (cyan). Scale bars 10 µm. (B) Same as (A) in MEF treated with BafA1. (C) Same as (B) in ATG5 KO MEF. (D) Same as (B) in ATG7 KO MEF. (E) Same as (B) in FAM134B KO MEF. F) Same as (B) in SEC62 KO MEF. (G) Same as (B) in STX17 KO MEF. (H) Same as (B) in FIP200 KO cells. (I) LysoQuant [24] quantification of HA-positive endolysosomes of A–H (*N* = 3 for C, D, F, G; *N* = 5 for A, B, E, *N* = 2 for H). Mean, *n* = 41, 85, 31, 12, 74, 29, 38, 44 cells. One-way ANOVA with Dunnett’s multiple comparisons test, ns *p* > 0.05, *p < 0.0001. (J) CLSM analyses in FAM134B KO MEF transiently co-transfected with FAM134B-HALO or FAM134BLIR-HALO (magenta) and NS_PL-HA (red). LAMP1-positive endolysosomes (cyan). Scale bars 10 µm. (K) LysoQuant quantification of HA-positive endolysosomes of J (*N* = 2). Mean, *n* = 33, 19 cells. Unpaired t test with Welch’s correction, ****p < 0.0001.

### NS_PL is an ERLAD client

Next, we used CLSM to monitor the delivery of NS_PL in the LAMP1-positive degradative compartment in a series of cell lines characterized by defective LC3 lipidation and dysfunctional autophagy (ATG5 and ATG7 KO), lacking the ERphagy receptors FAM134B or SEC62, lacking the SNARE protein STX17, which controls membrane fusion events between the ER and degradative endolysosomes [12,13,27], or lacking the FIP200 protein, a core component of the ULK kinase complex required for autophagosome formation [28–30] (Figure 2C–L).

LC3 lipidation is a defining step in selective autophagy pathways as it attaches LC3 to membranes and thereby allows autophagy receptors to link cargos to the forming autophagosome or to degradative endolysosomes [25,31]. To assess whether lysosomal delivery of NS_PL depends on the LC3 lipidation machinery, the folding-defective polypeptide was transiently expressed in MEF lacking ATG5 (Figure 2C) [32] or ATG7 (Figure 2D) [33], two essential components required for LC3 conjugation. Loss of either ATG5 or ATG7 markedly reduced the delivery of NS_PL to endolysosomes (Figure 2C,D, respectively, Figure 2I).

Next, to determine whether an ERphagy receptor mediates the delivery of NS_PL to endolysosomes for clearance, the folding-defective polypeptide was transiently expressed in MEF lacking the ERphagy receptors FAM134B (Figure 2E, CRISPR cells are described in [12]), or SEC62 (Figure 2F, CRISPR cells are described in [34]). We observed that deletion of FAM134B substantially inhibits endolysosomal delivery of NS_PL (Figure 2E,L), whereas deletion of SEC62 had no consequence (Figure 2,F,L). The substantial decrease of delivery of NS_PL to endolysosomes in STX17 KO MEF (Figure 2G,I CRISPR cells are described in [12]) confirms that fusion events between the ER and the endolysosomal membrane are required to deliver the ERLAD client from the ER lumen to the endolysosomal lumen.

To test whether the delivery of NS_PL to endolysosomes depends on phagophore formation and *macro*-ERphagy pathways, we monitored the fate of the folding-defective polypeptide in MEF depleted of FIP200. In FIP200 KO MEF, the delivery of NS_PL to endolysosomes remains unaffected (Figure 2H,I). Taken together, these data reveal that the lysosomal clearance of NS_PL does not require the intervention of double membrane autophagosomes. Rather, NS_PL is delivered to endolysosomes via FAM134B-driven, LC3-mediated transport as previously reported for another polymerization-prone, disease‑causing mutant polypeptide, the Z mutant form of alpha1-antitrypsin [12,13].

Finally, we determined whether reconstitution of the FAM134B receptor in FAM134B KO MEF restores the delivery of NS_PL within endolysosomes. To this end, cells were back-transfected with plasmids for expression of either the active form of the ERphagy receptor (FAM134B-HALO, Figure 2J, upper panels, 2K) or an inactive form of the receptor that cannot bind the autophagy protein LC3 upon mutation of the LC3-interacting region (FAM134BLIR-HALO, Figure 2J, lower panels, 2K). The analyses by CLSM revealed that only the expression of the active FAM134B-HALO efficiently rescues NS_PL delivery to endolysosomal compartments in FAM134B KO MEFs (Figure 2J,K), confirming the central role of this ERphagy receptor in ERLAD of NS_PL.

### NS_PL associates with the CNX:FAM134B ERLAD functional complex

We previously showed that the lectin chaperone CNX participates in supra-molecular functional complexes that segregate ERLAD clients in ER subdomains that will eventually deliver misfolded proteins to the lysosomal compartments for clearance [12,13]. To verify this for NS_PL, HEK293 cells were mock-transfected (Figure 3, lane 1), or transiently transfected with plasmids for expression of V5-tagged FAM134B (lane 2), NS_PL (lane 3), V5-tagged FAM134B and HA-tagged NS (lane 4), or V5-tagged FAM134B and HA-tagged NS_PL (lane 5). The ectopically expressed FAM134B was immunoisolated from cell lysates (Figure 3, upper panel, lanes 6–10), and the presence of CNX or of NS and NS_PL in the immunocomplex was verified by immunoblot with anti-CNX (middle panel) or anti-HA antibodies (lower panel). The results of this experiment confirm the preferential capture of NS_PL in the functional complex formed by CNX and FAM134B (Figure 3, lane 10, lower panel).

**Figure 3.**
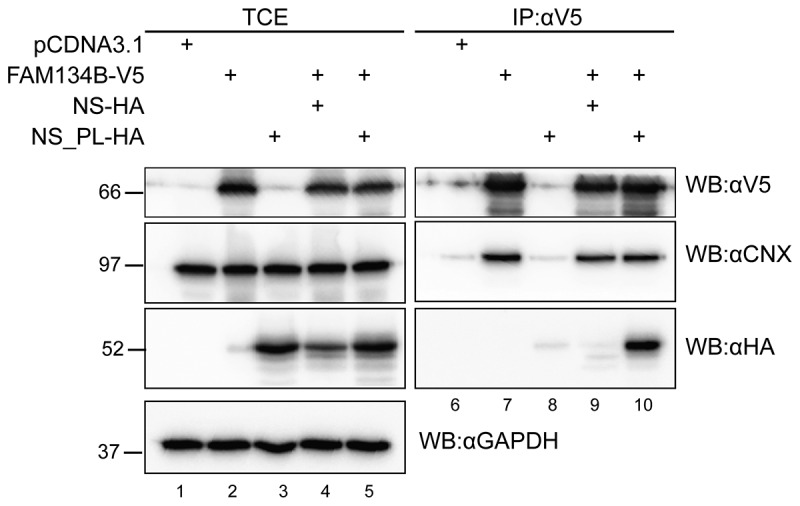
NS_PL associates with the CNX:FAM134B functional complex which then mediates ERLAD. HEK293 cells were transfected with empty vector (lanes 1, 6), FAM134B-V5 (2, 7), NS_PL-HA (3, 8), FAM134B-V5 and NS-HA (4, 9), or FAM134B-V5 and NS_PL-HA (5, 10), incubated for 6 h with 100 nM BafA1. Lanes 1–5, WB of the total cell extract (TCE); lanes 6–10, WB of anti-V5 immunoprecipitates to isolate complexes containing ectopically expressed FAM134B. The membranes were probed with anti-V5, anti-CNX, anti-HA.

### N-glycan processing channels NS_PL in the ERLAD machinery

The disease-causing NS_PL variant of NS is a di-glycosylated protein (the cryptic glycosylation site at position 401 is not modified in the NS and NS_PL protein, Figure 1A) [20].

*N*-linked glycans are rapidly processed by the α_1,2_-glucosidase I (GI in Figure 4A) that removes the outermost glucose 1, and by the α_1,2_-glucosidase II (GII) that removes glucose 2. These cleavages expose the innermost glucose 3 (circled in red in Figure 4A) which engages the lectin-like chaperone CNX [2]. GII also removes the innermost glucose 3 to disengage CNX. If the folding program has been completed, the mature polypeptide is released in the secretory line to be secreted from cells. If the protein is still unfolded, as it is the case for mutant proteins like NS_PL, the UDP-glucose:glycoprotein glucosyltransferase (UGGT1 in Figure 4A) re-glucosylates the *N*-linked oligosaccharides to prolong CNX engagement [2]. We previously reported that in the case of the Z-variant of alpha1-antitrypsin (ATZ) and of disease-causing variants of pro‑collagen, the sustained interaction with CNX drives the segregation of the terminally misfolded polypeptides into ER subdomains enriched in the ERphagy receptor FAM134B [12–14]. To assess whether the lysosomal clearance of NS_PL from the ER follows this route, the lysosomal delivery of the folding-defective protein was assessed by CLSM in MEF mock treated or subjected to pharmacologic inactivation of glycoprotein association with CNX. This was achieved by incubating cells with Castanospermine (CST), a cell permeable inhibitor of ER-resident GI and GII [2,11,35]. As shown in Figure 2, NS_PL accumulated within the LAMP1-positive compartments in MEF exposed to BafA1 (Figure 4B *vs*. 4C, 4G). Cells exposure to CST drastically reduced the lysosomal delivery of NS_PL (Figure 4D,G). Likewise, genetic inactivation of the CNX chaperone system upon deletion of the glucosylation enzyme UGGT1 [36,37] or upon deletion of CNX [38–40] substantially inhibited lysosomal delivery of NS_PL (Figure 4E,F, respectively, and 4G). Altogether these results show the importance of glucose processing and persistent lectin-engagement in FAM134B-driven NS_PL selection for ERLAD.

**Figure 4.**
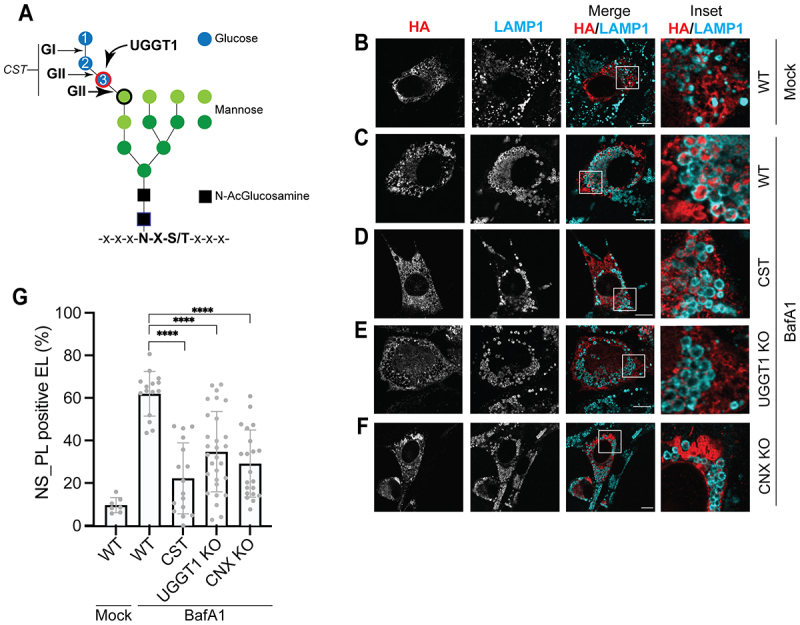
CNX and glucose processing enzymes regulate NS_PL delivery to endolysosomes. (A) N-glycan. Glucoses, blue; mannoses, green; N-acetylglucosamines, black. GI removes glucose 1. GII removes glucose residues 2 and 3. UGGT1 adds-back glucose 3, which engages CNX. CST inhibits GI and GII. (B) CLSM analyses in WT MEF. NS_PL-HA (red) in LAMP1-positive endolysosomes (cyan). Scale bars 10 µm. (C) Same as (B) in MEF treated with BafA1. (D) Same as (B) in MEF treated with BafA1 and CST. (E) Same as (C) in UGGT1 KO MEF. (F) Same as (C) in CNX KO MEF. (G) LysoQuant [24] quantification of endolysosomes containing the ERLAD client in (B-F). *N* = 3. Mean, *n* = 7, 15, 16, 29, 21 cells. One-way ANOVA with Dunnett’s multiple comparisons test, *p < 0.0001.

### The N-glycan at position 321 in NS_PL signals selection for ERLAD

NS_PL harbors three predicted *N*-glycosylation sites (N157, N321, and N401). Only those at position N157 and N321 are covalently modified with an *N*-linked oligosaccharide (green in Figure 5A) [20]. To confirm this, we performed partial digestions of NS and of NS_PL oligosaccharides using two different glycanases, EndoH (Figure 5B, upper panel) and Peptide-N-Glycosidase F (PNGaseF) (Figure 5B, lower panel). Western blot analyses revealed, with both assays, three distinct bands both for NS (Figure 5B, lane 2) and NS_PL (Figure 5B, lane 4) confirming that both NS and NS_PL carry two *N*-linked glycans. The lower polypeptide bands show the fully de-glycosylated NS and NS_PL polypeptides (arrows 0 NGly in Figure 5B, upper and lower panels). The middle bands show the partially de-glycosylated NS and NS_PL polypeptides (arrows 1NGly). The upper band is the unprocessed NS and NS_PL polypeptides (arrows 2NGly).

**Figure 5.**
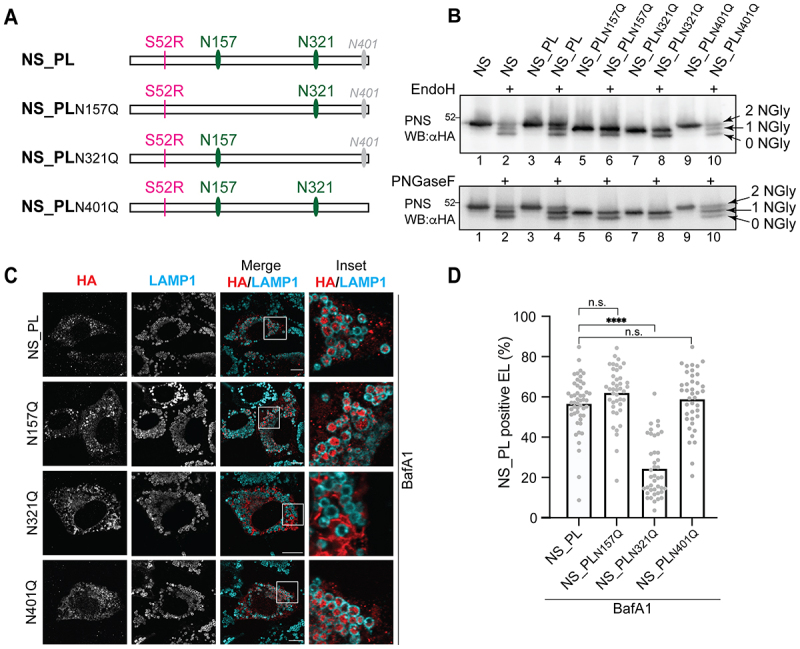
N-glycosylation at position 321 is essential for NS_PL delivery to endolysosomes. (A) NS_PL and its glycosylation site mutants. Glycosylation sites at N157 and N321 highlighted in green, the cryptic glycosylation site 401 is in grey. The S52R mutation of NS_PL is in pink. (B) Electrophoretic mobility of NS_PL and the glycosylation mutants expressed in HEK293 analyzed by SDS-PAGE and immunoblotting after partial EndoH or PNGaseF treatment (upper and lower panel, respectively). (C) CLSM analyses of NS_PL-HA and its glycosylation mutants (red) delivery within LAMP1-positive endolysosomes (cyan) in MEF WT cells treated with BafA1. Scale bars 10 µm. (D) LysoQuant [24] quantification of HA-positive endolysosomes of C. *N* = 3. Mean, *n* = 49, 42, 41, 40 cells. One-way ANOVA with Dunnett’s multiple comparisons test, ns *p* > 0.05, *p < 0.0001.

To confirm these data, we generated the glycosylation variants NS_PLN157Q, NS_PLN321Q, and NS_PLN401Q (Figure 5A) by replacing the asparagine (*N*) residue of the *N*-glycosylation sequons with a glutamine (Q) residue, thereby abolishing the modification of the respective sequons with pre-assembled oligosaccharides. The expectation was that NS_PLN157Q, NS_PLN321Q are mono-glycosylated polypeptides, whereas mutation of the asparagine residue at position 401 does not affect the di-glycosylation state of the protein. The partial EndoH and PNGaseF digestions reveal two polypeptide bands for NS_PLN157Q (Figure 5B, lane 6, arrows 1NGly and 0NGly) and NS_PLN321Q (Figure 5B, lane 8). This confirms that NS_PLN157Q and NS_PLN321Q carry only 1 *N*-linked oligosaccharide. The partial EndoH and PNGaseF digestions reveal 3 polypeptide bands for NS_PLN401Q showing that this form still carries 2 *N*-linked oligosaccharides (lane 10). These results confirm that NS_PL is glycosylated at positions N157 and N321 (Figure 5A, green ellipses), whereas the N401 is a cryptic glycosylation site (Figure 5A, gray ellipses).

To determine which, if any, of the NS_PL glycans functions as a signaling glycan for ERLAD, we monitored the lysosomal delivery of NS_PL and of the glycosylation mutants NS_PLN157Q, NS_PLN321Q and NS_PLN401Q in transiently transfected MEF treated with BafA1. CLSM reveals that deletion of the glycosylation site at position 157 or of the cryptic glycosylation site at position 401 does not affect lysosomal delivery of NS_PL (Figure 5C,D). In sharp contrast, the deletion of the glycosylation site at position 321 substantially hampers the delivery of the misfolded polypeptides within LAMP1-positive endolysosomes (Figure 5C,D). Thus, the *N*-glycan at position 321 is required for lysosomal delivery of terminally misfolded NS_PL aggregates. These observations were consistent with an increased accumulation of NS_PL_N321Q in the detergent-insoluble fraction of HEK293 cells expressing NS_PL and its glyco-mutants, as determined by SDS–PAGE followed by immunoblotting (Figure 6A,D). Additionally, the secretion of all glyco-mutants was comparably low and consistently reduced relative to the wild-type NS protein (Figure 6A,B).

**Figure 6.**
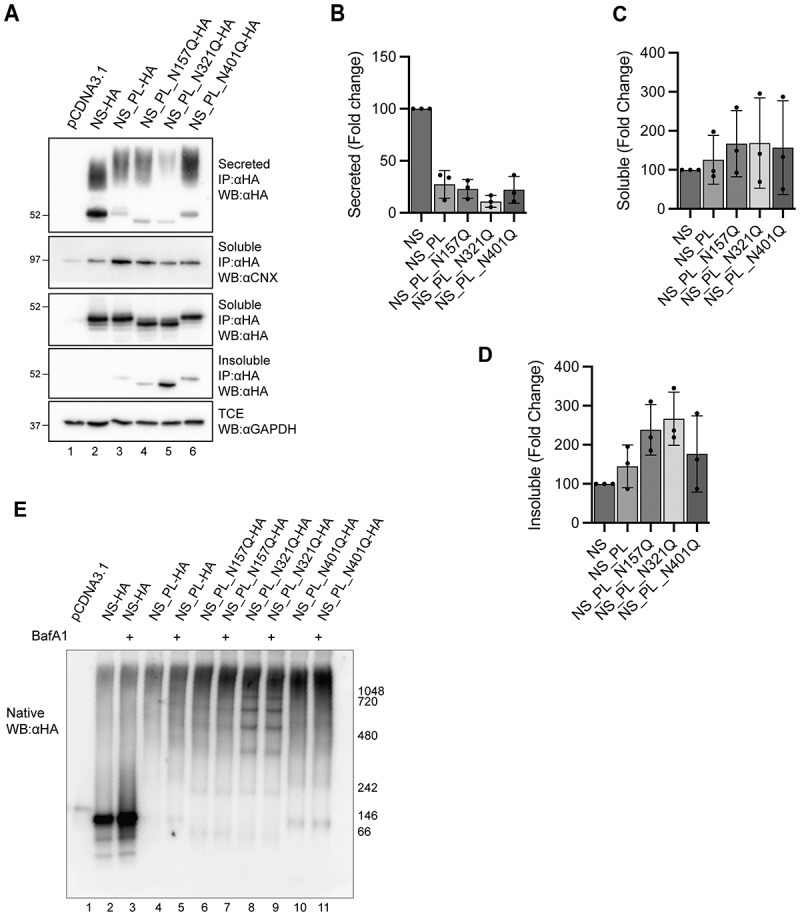
Impaired interaction of NS_PL _N321Q with CNX correlates with defective ERLAD and increases insoluble aggregation. (A) HEK293 cells were transiently transfected with NS-HA or NS_PL-HA. NS-HA and NS_PL-HA were immunoisolated with anti-HA antibodies from distinct cellular fractions (secreted, detergent-soluble, and detergent-insoluble). Proteins were separated in SDS-PAGE and revealed by immunoblotting. (B) Quantification of the secreted fraction of A, *N* = 3. (C) Same as (B) but for the detergent-soluble fraction. (D) Same as (B) but for the detergent-insoluble fraction. (E) HEK293 cells were transiently transfected with NS-HA or NS_PL-HA and mock-treated or exposed to 100 nM BafA1 and analyzed by nondenaturing PAGE and immunoblotting.

The immunoprecipitation of HA-tagged NS_PL resulted in the co-precipitation of endogenous CNX, as detected by immunoblotting. This interaction was substantially reduced for NS_PL_N321Q (Figure 6A), indicating that the glycan at position N321 plays an important role for the interaction with CNX. Moreover, non-denaturing PAGE analysis of the same samples revealed that the removal of the *N*-glycan at position 321 resulted in accumulation of high-molecular-weight polymers as a further indication of dysfunctional selection of this mutant for ERLAD (Figure 6E).

Thus, these findings support a model in which glucose processing (removal by GII and re-addition by UGGT1) of a specific *N*-glycan on folding-defective, polymerogenic ERLAD clients sustains CNX interaction, engagement of the ERphagy receptor FAM134B, and lysosomal clearance.

## Discussion

Large, misfolded proteins and misfolded polypeptides that form aggregates or polymers cannot be dislocated across the ER membrane for proteasomal clearance. Rather, they are segregated in ER subdomains that deliver their toxic content within degradative endolysosomes for clearance via *macro*-ERphagy, *micro*-ERphagy, or LC3-dependent delivery programs, or are extracted from the ER and subsequently delivered within degradative endolysosomes in catabolic programs that are collectively named ER-to-Lysosome Associated degradation (ERLAD) [5–7]. In recent years, ERLAD pathways have been described for an increasing number of clients for which the lysosomal clearance relies on the intervention of specific ERphagy receptors. For example, ATZ polymers, mutant forms of Pro-collagen or of Niemann–Pick type C protein are processed through FAM134B-dependent mechanisms, whereas other aggregation-prone proteins such as secretory acinar enzymes or six-repeat islet amyloid polypeptide engage CCPG1, and misfolded proinsulin or pro-arginine vasopressin, and proopiomelanocortin (POMC) rely on RTN3L-mediated ERphagy [6,7].

The processing of *N*-linked oligosaccharides determines the fate of the associate polypeptide. Sparse and rare reports show that a single *N*‑glycan within multi‑glycosylated proteins can execute a dominant role in *N*‑glycan-regulated folding [41], *N*‑glycan-dependent selection for ERAD [42] or *N*‑glycan-dependent selection for ERLAD [13]. Regarding protein folding, Ari Helenius and colleagues showed that only the glycan at position 81 among the seven *N*‑linked oligosaccharides on influenza virus hemagglutinin (HA) is strictly required for HA maturation [41]. Similarly, for ERAD, Spear and Ng found that during clearance of folding defective, mutant forms of the tetra‑glycosylated CPY and of the di‑glycosylated PrA proteins from the yeast ER, only the glycans at position 479 of CPY and at position 107 of PrA are necessary to direct these misfolded polypeptides into the ERAD pathway [42]. In our previous work, by tracking the fate of misfolded polymers of the tri-glycosylated Z-variant of alpha1-antitrypsin, we demonstrated that the *N*-linked glycan at position 83 is crucial for the persistent engagement of CNX, which ensures the FAM134B-mediated ERLAD of this disease-causing polypeptide [13]. Also, persistent glucose processing of the *N*-glycan at position 1388 of a disease-causing mutant form of Pro-collagen2A1 selects this large misfolded polypeptide for lysosomal delivery [13].

With this study, we expand the repertoire of ERLAD clients that require processing of a specific *N*-linked oligosaccharide for efficient clearance from the ER lumen. For the aggregation-prone, FENIB-causing NS_PL, delivery within LAMP1-positive degradative endolysosomes is markedly impaired following either pharmacologic or genetic inactivation of the enzymes responsible for removing and re-adding terminal glucose residues on protein-bound *N*-linked oligosaccharides as well as upon depletion of CNX, the lectin chaperone that mediates the interaction of mono-glucosylated ERLAD clients with the ERphagy receptor FAM134B [12]. Among the two N-linked oligosaccharides present on NS_PL, only the glycan at position 321 is required for efficient trafficking of the aberrant polypeptide from the ER lumen to degradative endolysosomes. The presence of this oligosaccharide strengthens the association of NS_PL with CNX. Although we did not directly assess its glucosylation state, these findings support the conclusion that the glycan at position 321, its processing by resident glucosidases and glucosyltransferases and the enhanced engagement of CNX that it promotes, are all critical determinants of NS_PL selection for FAM134B-mediated ERLAD.

In contrast to the lysosomal clearance of mutant, folding-defective pro-Collagen2A1, which depends on genes that regulate autophagosome biogenesis (e.g., FIP200) and proceeds via the *macro-*ERphagy branch of ERLAD [14], FIP200 and *macro*-ERphagy are dispensable for the clearance of NS_PL. Instead, lysosomal delivery of NS_PL requires the coordinated action of the ER lectin CNX in complex with the ERphagy receptor FAM134B, components of the LC3 lipidation machinery, and the SNARE protein STX17. This clearance program closely mirrors the LC3-dependent delivery program previously described for the lysosomal disposal of ATZ polymers [12]. Collectively, these findings strengthen the concept that individual *N-*glycans serve as signaling appendages that dictate protein fate, not only for protein folding/secretion and ERAD, but also during ERLAD selection [13,41,42]. A key open question is what structural features render specific *N*-glycans dominant in determining a polypeptide’s fate. One plausible explanation is their position within regions of distinct structural or dynamic properties, such as rigid or flexible loops or locally mobile segments. These environments likely enhance accessibility to ER-resident enzymes (glucosidases, mannosidases, and glucosyltransferases), and to mannose- (for ERAD selection) or glucose-binding lectins (for ERLAD selection). In this scenario, de-glucosyated *N-*glycans on properly folded polypeptides engage cargo receptors such as ERGIC53, VIP36, or VIPL lectins at ER exit sites, promoting secretion [2]. De-mannosylated *N*-glycans on terminally misfolded proteins recruit the ER-resident mannose-binding lectins OS-9 and XTP3-B directing the substrate to the cytoplasm for ubiquitin-proteasome degradation via ERAD [2]. Persistently glucosylated *N*-glycans on large, misfolded polypeptides engage glucose-binding lectins such as CNX or calreticulin. These lectins form functional complexes with ERphagy receptors, thereby segregating toxic conformers into specialized ER subdomains that are eventually separated from the bulk ER and/or deliver aberrant gene products to degradative endolysosomal compartments in processes collectively defined as ERLAD [43].

## Materials and methods

### Cell culture, transient transfection, and inhibitors

MEF and HEK293 were grown in DMEM supplemented with 10% FBS at 37°C and 5% CO2. Atg7 (WT and KO) and Atg5 (WT and KO) MEF were gifts from M. Komatsu and N. Mizushima. CNX KO [38–40] and UGGT1 KO MEF [36,37] have been previously described. SEC62, FAM134B, and STX17 KO MEF cells were engineered using CRISPR/Cas9 system as previously described [12,34]. Transient transfections were performed using JetPrime transfection reagent (PolyPlus, 101,000,001) following manufacturer’s protocol. BafilomycinA1 (InvivoGen, tlrl-baf1) was used at 50 nM for 12 h or 100 nM for 6 h. CST (Sigma, TA9H9A9AC0B2-5 MG) and was used at 1 mM.

### Confocal laser scanning microscopy (CLSM)

MEFs were plated on pre-coated Alcian blue (Sigma, A5268) glass coverslips (Biosystems, 0117520) prior to transient transfection using the jetPRIME reagent following the manufacturer’s protocol. For LysoQuant analysis, cells were treated with 50 nM BafA1 for 17 h. For samples with a HaloTag 100 nM TMR (Promega, G8251) was added together with BafA1. 24 h post-transfection, cells were fixed at room temperature for 20 min in 3.7% paraformaldehyde (Sigma, 8,187,081,000) in PBS. After three washes with PBS coverslips were then incubated for 20 min in saponin permeabilization solution (PS) (10% goat serum, 10 mM HEPES, 15 mM glycine, and 0.05% saponin in PBS) to allow antibody access to intracellular structures. Following permeabilization, cells were incubated with primary antibodies diluted in PS for 90 min. After three washes with PS, cells were incubated with Alexa Fluor – conjugated secondary antibodies diluted 1:300 in PS for 45 min. Finally, cells were rinsed three times with PS and water and mounted onto glass microscope slides (Epredia, 4941–8515-001T) using VECTASHIELD (Vector Laboratories, H-1200–10) supplemented with 4,’6-diamidino-2-phenylindole (DAPI). Confocal images were captured using STELLARIS 5 and STELLARIS 8 microscopes equipped with a Leica HCX PL APO lambda blue 63×/1.40 oil objective and a pinhole set at 1 a.u. Image acquisition was performed with Leica LAS X software, utilizing diode at 405 nm or laser beams at 489, 499, 552, 561, 587 and 653 nm wavelengths for excitation. Fluorescence emissions were collected at the following ranges: 430–490 nm (Alexa Fluor 405), 504–587 nm (Alexa Fluor 488), 592–644 nm (Alexa Fluor 568) and 658–750 nm (Alexa Fluor 646). Image analysis and quantification were performed using LysoQuant and ImageJ 2.16.0/1.54p software64,123. Image post-processing was performed with Adobe Photoshop (v26.2.0).

### Cell lysis, immunoprecipitation, and western blot

After the respective treatments, cells were washed with ice-cold phosphate-buffered saline (PBS) containing 20 mM N-ethylmaleimide (NEM). The cells were then lysed using either 2% CHAPS (in HEPES-buffered saline (HBS), pH 6.8) or RIPA buffer (1% Triton X-100, 0.1% SDS, 0.5% sodium deoxycholate in HBS, pH 7.4), both supplemented with 20 mM NEM and protease inhibitors (200 mM phenylmethylsulfonyl fluoride, 16.5 mM chymostatin, 23.4 mM leupeptin, 16.6 mM antipain, 14.6 mM pepstatin) for 20 min on ice. Post-nuclear supernatants (PNS) were collected after centrifugation at 10,600 g for 10 min. The PNS was denatured by adding 100 mM dithiothreitol (DTT) and heated for 5 min at 95°C before being subjected to SDS–polyacrylamide gel electrophoresis (PAGE). Detergent insoluble material was solubilized in 8 M Urea and dissolved in 0.1 M ammonium bicarbonate for further analyses. For immunoprecipitations, PNSs were diluted with lysis buffer and incubated with Protein G beads (1:10 w/v, swollen in PBS) and select antibodies at 4°C. After three washes of the immunoprecipitants with 0.5% CHAPS in HBS pH 7.4, beads were denatured for 5 min at 95°C and subjected to SDS–PAGE. Proteins were transferred to PVDF membranes using the Trans-Blot Turbo Transfer System (Bio-Rad, 17,001,915). Native gel electrophoresis was performed after CHAPS lysis. The PNSs were incubated for 15 min at RT in Native Sample Buffer (Bio-Rad, 1610738EDU) and run on 7.5% native acrylamide gel in Tris/Glycine Buffer (Bio-Rad, 1610771EDU). Proteins were then transferred onto PVDF membrane using the Trans-Blot Turbo Transfer System (Bio-Rad, 17,001,915). Membranes were blocked with 10% (w/v) nonfat dry milk (Bio-Rad, 10,004,504) in Tris-buffered saline containing 1% Tween 20 (TBS-T; Sigma-Aldrich, 93,362, 71,376, H1758, P9416) and stained with primary antibodies diluted in TBS-T. Afterward, horseradish peroxidase (HRP)-conjugated protein A or HRP-conjugated secondary antibodies diluted in TBS-T were applied for 45 min. Membranes were developed using Western Bright ECL and signals captured on Fusion FX Edge 18.12 (Vilber). The quantification of western blot bands was performed using Fusion FX Edge 18.12 and ImageJ 2.16.0/1.54p software123. Membrane stripping for probing additional antigens was done using Re-Blot Plus Strong Solution (Millipore, 2504) following manufacturer’s instructions.

### EndoH and PNGaseF assays

For full EndoH (NEB, P0702S) and PNGaseF (NEB, P0704S) treatment, proteins from immunoprecipitated samples or from TCE were split into two aliquots and incubated in the presence or absence of 5 mU of EndoH for 3 h at 37°C according to the manufacturer’s protocol. For partial EndoH and PNGaseF assays, samples were instead incubated with 2.5 mU of enzyme for 10 min at 37°C. Samples were then analyzed by SDS–PAGE.

### Expression plasmids and antibodies

NS-HA, NS_PL-HA, NS_PL_N157Q-HA, NS_PL_N321Q-HA, and NS_PL_N401Q-HA expression plasmids were prepared by subcloning the coding sequence of NS variants (ordered by GeneScript) with a C-terminal HA tag in pcDNA3.1(+).

FAM134B-Halo and FAM134BLIR-Halo (DDFELL to AAAAAA) expression plasmids were prepared by subcloning the FAM134B in a pcDNA3.1(+) backbone with a C-terminal HALO-tag. FAM134B-V5 is subcloned in pcDNA3.1(+) and was prepared as previously described [12].

Commercial antibodies used in this study were against polymeric ATZ (2C1, HycultBiotech), GAPDH (Merck, MAB374), HA (Sigma, H6908), LAMP1 (Hybridoma Bank, 1D4B was deposited to the DSHB by August, J.T.), Giantin (Biolegend, 920,342), V5 (Thermo Fisher, R96025). Anti-CNX was a kind gift from A. Helenius.

Alexa-conjugated secondary antibodies were purchased from Thermo Fisher Scientific, HRP-conjugated from Jackson ImmunoResearch, and Protein A-conjugated from Invitrogen.

## Statistical analysis

The statistical comparisons and graphical plots were performed using GraphPad Prism 11 (GraphPad Software, Version 11.0.2 (100) for MAC). Tests employed for statistical analysis are reported in figure legends. Data distributions (individual points) are always shown. An adjusted *p* value < 0.05 (for one-way and two-way analysis of variance (ANOVA) with multiple comparison tests) was considered statistically significant. Results from ANOVA analyses were adjusted for multiple comparisons where appropriate. All t-tests were performed as two-sided tests. All experimental replicates represent independent biological replicates. The corresponding figure legends indicate the number of cells analyzed (n) and the number of independent biological replicates (N). Quantification of fluorescent images assessing HA delivery to endolysosomes was performed using LysoQuant [24], an automated and unbiased deep learning-based analysis tool.

## Supplementary Material

Rawdata.xlsx

## Data Availability

The authors confirm that the data supporting the findings of this study are available within the article and its supplementary materials.

## Abbreviations

BafA1: Bafilomycin A1
CLSM: Confocal Laser Scanning Microscopy
CST: Castanospermine
CNX: Calnexin
EndoH: Endoglycanase H
ER: Endoplasmic Reticulum
ERAD: ER-Associated Degradation
ERLAD: ER-to-lysosome Associated Degradation
GI: α-glucosidase I
GII: α-glucosidase II
NS: Neuroserpin
NS_PL: Neuroserpin Portland mutation (S52R)
PNGaseF: Peptide-N-Glycosidase F
UGGT1: UDP-glucose:glycoprotein glucosyltransferase
STX17: Syntaxin17
